# Membrane Oscillations Keep Neurons on the Right Track

**DOI:** 10.1371/journal.pbio.0040191

**Published:** 2006-05-16

**Authors:** Liza Gross

Whether you are asleep or awake, engaged in flights of fancy, or engrossed in intense concentration, your brain is a hotbed of electrical and chemical activity. Nerve cells have channels that regulate the flow of ions in and out of their membrane. In a resting neuron, the potential inside the cell is negative, averaging about −70 millivolts, compared with the outside. Signals generated by a sensory stimulus drive the membrane potential to a less negative, depolarized, threshold value (about −55 millivolts). The neuron then releases a stream of electrical activity—as series of action potentials, or spikes—along its membrane. After firing an action potential, the membrane potential becomes even more negative than when at rest (called hyperpolarization), and the neuron resists firing for a variable period of time. The timing and firing rate of such spikes are all the information the brain has to represent a given sensory stimulus.

All the while neurons should be conveying precise signals, they are exposed to various sources of electrical noise. This noise causes “jitter”—tiny fluctuations in the timing of any single action potential with respect to the signal that can accumulate and affect the accuracy of a transmitted message. On top of this noise, a neuron's resting potential oscillates around the resting value, well below the action potential threshold. Interestingly, and counterintuitively, increasing evidence suggests that this additional variability may help neurons separate signal from noise. In a new study, Troy Margrie and colleagues have used a combination of experimental and theoretical approaches to investigate the role of membrane potential oscillations (MPOs) in signal processing. They show that oscillations in a single cell improve the precision of an action potential by reducing the accumulation of jitter that is inherent during ongoing spiking.

It has been suggested that spike sequences (called spike trains) in hippocampal and olfactory cells adjust their timing to the phase of the cells' MPOs, thereby providing action potentials with a frame of reference that enhances signal processing. MPOs in the olfactory system are tied to the breathing cycle. To artificially manipulate the electrical signals in individual neurons and determine how MPOs affect action potential precision, the researchers recorded intracellularly from individual neurons in double tracheotomized mice. Since the overall timing of action potentials was the same for tracheotomized as compared with free-breathing mice, this meant the researchers could use the tracheotomized animals to compare action potential precision under oscillatory and nonoscillatory (control) conditions. They found that oscillations “greatly enhanced” the overall precision of the action potentials—though precision decreases with the number of preceding action potentials within an oscillation cycle.

To further investigate the source of this precision, the researchers recorded from mitral cells in olfactory bulb slices and explored the mechanism of enhanced precision by introducing alternating pulses of depolarizing and hyperpolarizing currents to simulate the natural oscillation cycle. Jitter accumulated with long periods of depolarization, but when a hyperpolarizing current was delivered between two action potentials, the second spike recovered the precision of the first. Longer hyperpolarizing periods “dramatically reduced” the variance of the membrane potential—and yielded the most precise action potentials. These results support their in vivo evidence that spike trains within an oscillation cycle lose their enhanced precision since the trough of the oscillation is absent during such a spike train.

With enhanced precision comes enhanced performance. Exposing different types of neurons to over 77 stimulus pairs showed that MPOs significantly increased a neuron's ability to distinguish temporally distinct stimulus patterns. Using simulations to vary the frequency and amplitude of the oscillations and the kinetics of the stimulus currents, the researchers found enhanced stimulus discrimination over “physiologically realistic ranges” of firing rates, input kinetics, and oscillation parameters. For low oscillation frequencies, however, stimulus discrimination is most robust when synaptic inputs arrived at the trough and early rising phase of an oscillation cycle.

By establishing a hyperpolarizing period, oscillations prevent jitter accumulation, which improves action potential precision and stimulus discrimination. That this feature occurs in different types of neurons suggests a relatively simple and general underlying mechanism, the researchers argue. Any system where inputs must be integrated and transformed into discrete outputs, from circadian rhythms to gene expression programs, would likely benefit from any reset mechanism that minimizes the accumulation of noise.

## 

**Figure:**
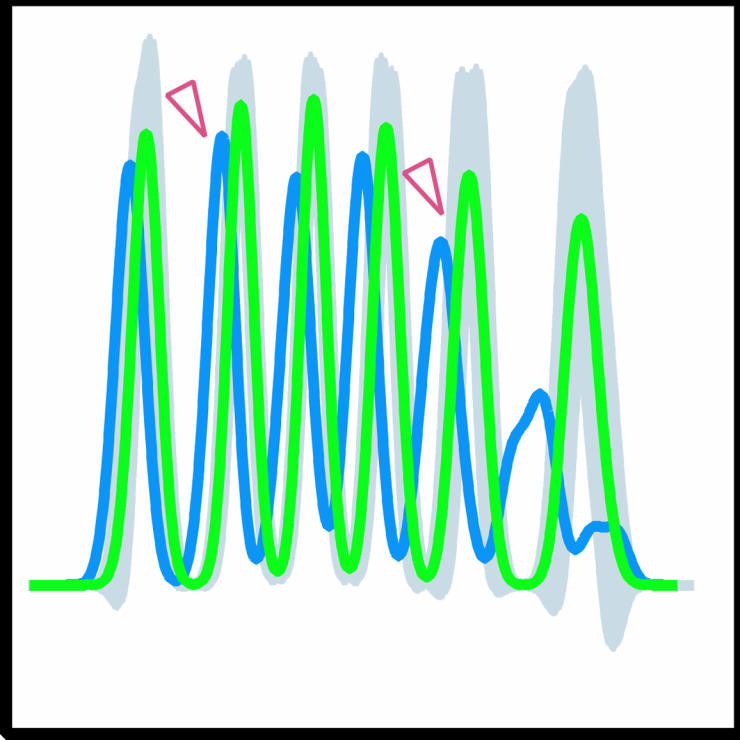
A mean peri-stimulus time histogram for two stimuli (blue and green) in the presence of a background oscillation. The gray shading represents the variance of the green stimulus; oscillations permit the discrimination of stimulus-specific situations (red arrows).

